# Oxytocin exerts harmful cardiac repolarization prolonging effects in drug-induced LQTS

**DOI:** 10.1016/j.ijcha.2022.101001

**Published:** 2022-04-03

**Authors:** Paul Kreifels, Ilona Bodi, Tibor Hornyik, Gerlind Franke, Stefanie Perez-Feliz, R. Lewetag, Robin Moss, Alessandro Castiglione, David Ziupa, Manfred Zehender, Michael Brunner, Christoph Bode, Katja E. Odening

**Affiliations:** a Department of Cardiology and Angiology I, University Heart Center Freiburg, Medical Faculty, University of Freiburg, Freiburg, Germany; b Institute for Experimental Cardiovascular Medicine, University Heart Center Freiburg • Bad Krozingen, and Faculty of Medicine, University of Freiburg, Freiburg, Germany; c Translational Cardiology, Department of Cardiology, Inselspital, University Hospital Bern, and Institute of Physiology, University of Bern, Bern, Switzerland; d Department of Cardiology and Medical Intensive Care, St. Josef Hospital, Freiburg, Germany

**Keywords:** Acquired long-QT syndrome, Arrhythmia mechanisms, Drug induced QT-prolongation, Ion channels

## Abstract

**Background:**

Oxytocin is used therapeutically in psychiatric patients. Many of these also receive anti-depressant or anti-psychotic drugs causing acquired long-QT-syndrome (LQTS) by blocking HERG/I_Kr_. We previously identified an oxytocin-induced QT-prolongation in LQT2 rabbits, indicating potential harmful effects of combined therapy. We thus aimed to analyze the effects of dual therapy with oxytocin and fluoxetine/risperidone on cardiac repolarization.

**Methods:**

Effects of risperidone, fluoxetine and oxytocin on QT/QTc, short-term variability (STV) of QT, and APD were assessed in rabbits using *in vivo* ECG and *ex vivo* monophasic AP recordings in Langendorff-perfused hearts. Underlying mechanisms were assessed using patch clamp in isolated cardiomyocytes.

**Results:**

Oxytocin, fluoxetine and risperidone prolonged QTc and APD in whole hearts. The combination of fluoxetine + oxytocin resulted in further QTc- and APD-prolongation, risperidone + oxytocin tended to increase QTc and APD compared to monotherapy. Temporal QT instability, STV_QTc_ was increased by oxytocin, fluoxetine / fluoxetine + oxytocin and risperidone / risperidone + oxytocin. Similar APD-prolonging effects were confirmed in isolated cardiomyocytes due to differential effects of the compounds on repolarizing ion currents: Oxytocin reduced I_Ks_, fluoxetine and risperidone reduced I_Kr_, resulting in additive effects on I_Ktotal_-tail. In addition, oxytocin reduced I_K1_, further reducing the repolarization reserve.

**Conclusion:**

Oxytocin, risperidone and fluoxetine prolong QTc / APD. Combined treatment further prolongs QTc/APD due to differential effects on I_Ks_ and I_K1_ (block by oxytocin) and I_Kr_ (block by risperidone and fluoxetine), leading to pronounced impairment of repolarization reserve. Oxytocin should be used with caution in patients in the context of acquired LQTS.

## 1 Introduction

Congenital long QT syndrome (LQTS) – a rare genetic channelopathy with increased risk for lethal ventricular arrythmias – is caused by mutations in cardiac ion channels [1], [2]. A similar, but more frequent form of QT prolongation, the acquired LQTS, is caused by a variety of drugs, which block repolarizing potassium currents, and shows similar clinical features (prolonged QTc and ventricular arrhythmias) as the inherited form [3]. I_Kr_ current, conducted by HERG channels, is a key outward potassium current in phase-3-repolarization of the cardiac action potential and the main target for drugs causing acquired LQTS [4]. Antipsychotics, such as risperidone, as well as antidepressants, such as fluoxetine, block I_Kr_ potentially leading to severe cardiac events such as torsade-de-pointes tachycardia or even sudden cardiac death [5], [6].

Patients suffering from autism spectrum disorder and borderline personality disorder often receive drug treatment with risperidone or fluoxetine [7]. As an inherited impairment of the oxytocin system - that affects the brain with regard to cognitive and behavioral processes - is thought to be a key aspect regarding the etiology of these diseases [8], novel combined medical therapies with oxytocin are used in those patients. Oxytocin exerts anxiolytic effects, attenuates behavioural processes towards stress and promotes social functions such as trust, the so-called “bonding effect” [9], [10]. Oxytocin, however, has also demonstrated QT-prolonging effects in human and animal studies [11], [12], [13], [14]. We could previously demonstrate QT-prolonging and pro-arrhythmic effects in transgenic LQT2 rabbits [15].

In the current study, we aimed at elucidating potential QT-/APD-prolongation and resulting pro-arrhythmia indicators by combined treatment with oxytocin and risperidone or fluoxetine in wild-type rabbits, their explanted hearts and single cardiac myocytes. The rabbit was chosen as model system due to pronounced species similarities in cardiac repolarizing ion currents between humans and rabbits [16] that facilitate future translation to human subjects.

## 2 Methods

A more detailed method description can be found in the online supplement.

### 2.1 Animal studies

All animal experiments were performed in accordance with the German animal protection law (TierSchG) and the Directive 2010/63/EU of the European Parliament after approval by the local authorities (Regierungspräsidium Freiburg; protocol number G15/114). As female subjects and animals are more susceptible to drug-induced, acquired LQTS [17] only adult female wild type *New Zealand White* rabbits of similar age and weight were used for *in vivo* and *ex vivo* experiments.

For sedation, S-Ketamine (12.5 mg/kg, Pfizer, USA) and xylazine (3.75 mg/kg, Bayer, Germany) were administered i.m. for surface ECG and MAP experiments and maintained by continuous i.v. infusion of ketamine/xylazine (2.5–5 ml/kg/h) when performing surface ECG experiments, as this combination does not influence myocardial repolarization [18]. For monophasic action potential measurements, beating hearts were excised after additional application of sodium heparin (1.000 IU i.v. Braun, Germany) and sodium thiopental (40 mg/kg i.v., Inresa, Germany).

### 2.2 Surface ECG

Surface ECG measurements were performed in sedated WT rabbits at baseline and with oxytocin (bolus 1.5 IU i.v., infusion with 1 IU/ml at a rate of 6 ml/h), with fluoxetine (bolus 1 mg/kg i.v., infusion with 2 mg/ml at a rate of 4.5 ml/h) and risperidone (bolus 0.3 mg/kg i.v., infusion with 0.3 mg/ml at a rate of 10 ml/h). As we have previously demonstrated stable QTc values in temporal “sham” control measurements over > 30 min [15], no temporal control at baseline conditions were performed. I.v.-injection was used to ensure adequate blood concentration of the different compounds and to avoid uncertainties in serum concentrations following oral administration that may stem from species differences in pharmacokinetics.

RR-interval, QT duration, heart-rate corrected QTc (using the Fridericia formula $QTc=QT\;/\;\sqrt[{3}]{RR\;}/s$) and short-term variability of QT (STV_QT_) [19], a marker for temporal heterogeneity of repolarization, were assessed. For STV_QT_, 31 consecutive QT were measured and STV_QT_ was calculated using the following equation: STV_QT_ = ∑|D_n+1_ − D_n_| (30×√2)^−1^, where D is QT duration.

### 2.3 Monophasic action potential measurements (MAP)

To assess cardiac action potential (AP) duration, monophasic action potential measurements (MAP) were performed *ex vivo* (as described in [15]) at baseline and during drug infusion with oxytocin (200 ng/l), fluoxetine (3 µM), or risperidone (1 µM) and their combination with oxytocin at 2 Hz, 3 Hz and 4 Hz stimulation. For analysis, Isoheart® Software Version 1.1.1.128 was used (Hugo Sachs Electronic, Germany).

### 2.4 Patch clamping

#### 2.4.1 Isolation of rabbit ventricular cardiomyocytes

Standard collagenase digestion was used to isolate ventricular cardiomyocytes [18]. After anesthesia and euthanasia as described above, rabbit hearts were rapidly excised, mounted on a Langendorff apparatus, and perfused with oxygenated, 37 °C warm Tyrode solution supplemented with 0.8–1 mg/mL collagenase (Worthington type 2) and 33 µM Ca^2+^ for 25–40 min.

#### 2.4.2 Electrophysiological recording in rabbit cardiomyocytes

To assess the effects of the compounds on AP and ion currents, patch clamp experiments were performed using Axopatch 200B patch clamp amplifier (Molecular Devices) and pCLAMP software (Axon Instruments).

APs were recorded in current clamp with 2–3 ms 0.4 mA square pulses at 0.5, 1 and 2 Hz. Membrane currents were recorded using the whole cell configuration. For studies of I_K_ (I_Kr_ + I_Ks_), and I_K1_ cardiomyocytes were superfused with normal Tyrode solution. To measure I_Kr_, I_Ca,L_ and I_Ks_ were inhibited by 1 µM nisoldipine (Sigma) and 30 µM chromanol 293B (Sigma). *I-V* relationship for I_K_ and I_Kr_ tail currents were determined by applying 1.5 s depolarizing voltage pulses from holding potential of −40 mV to test potentials ranging from −30 to + 60 mV. Tail current was measured upon repolarization to −40 mV.

I_K1_ was elicited from a holding potential of −20 mV by voltage steps from −120 mV to + 50 mV. Steady-state I_K1_ amplitudes were estimated at the end of the 500 ms pulse. All experiments were carried out at room temperature.

## 3 Results

### 3.1 Surface ECG

To assess *in vivo* effects of oxytocin and its combination with psychopharmaceutic drugs on QT intervals, surface ECG were measured.

#### 3.1.1 Changes in QT and RR over time

Oxytocin (n = 13), fluoxetine (n = 23), risperidone (n = 21) and their combination with oxytocin all prolonged QT duration compared to baseline (Suppl. Fig. 1A-C) – with a more pronounced QT prolongation upon combined exposure. As heart rate slowed during the experiments (particularly during oxytocin (Suppl. Fig. 1A-C) but also during fluoxetine perfusion (Suppl. Fig. 1B)), heart-rate corrected QT durations were calculated (QTc).

#### 3.1.2 QTc duration

**Fig. 1:**
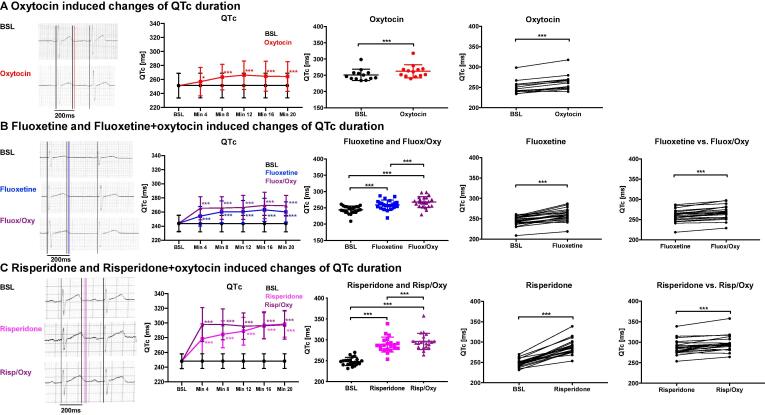
**Drug and hormone effects on heart-rate corrected QTc duration.** Oxytocin (n = 13) (**A**), fluoxetine and fluoxetine + oxytocin (n = 23) (**B**), risperidone and risperidone + oxytocin (n = 21) (**C**). Exemplary ECG recordings are shown in left columns. Indicated are QT intervals at baseline (black lines) and with hormones / drugs (colored lines). Middle columns: Indicated are changes over time in QTc during hormone / drug perfusion and dots plots of individual QTc at baseline at 8 min post bolus and with hormone / drug perfusion. Right column: Individual changes of QTc between baseline and hormone / drug treatment. Differences are indicated as * p < 0.05; ** p < 0.01; ***p < 0.001.

Application of oxytocin resulted in a significant increase of mean QTc duration compared to baseline in WT (Fig. 1A; comparison at 20 min after exposure). Similarly, fluoxetine (Fig. 1B), risperidone (Fig. 1C) or their combination with oxytocin significantly prolonged QTc. Important to note, a significant difference between the QT-prolonging effect of fluoxetine alone and fluoxetine + oxytocin (fluo + oxy) and between risperidone alone and risperidone + oxytocin (risp + oxy) was observed (Fig. 1) with a further prolongation in the combined treatment. On the right-hand side of Fig. 1A-C the individual prolongation of QTc duration compared to baseline is shown for each individual rabbit, indicating that this prolongation is seen among all animals.

#### 3.1.3 Short term variability (STV)

**Fig. 2:**
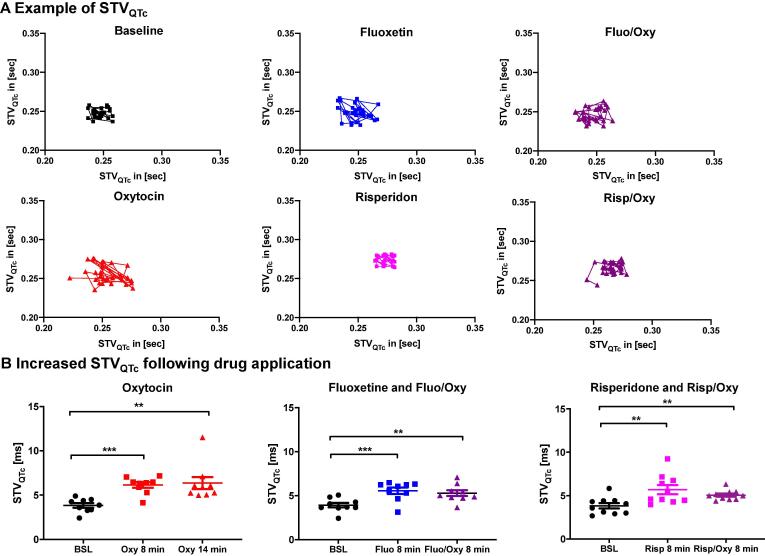
**Drug and hormone effects on short-term variability of QTc (STV_QTc_). A.** Examples of Pointcaré plots indicating STV_QTc_ at baseline, and during administration of oxytocin, fluoxetine, fluoxetine + oxytocin, risperidone, and risperidone + oxytocin. **B.** Dot plots indicating changes in STV_QTc_ following application of oxytocin (n = 9), fluoxetine and fluoxetine + oxytocin (n = 9), risperidone and risperidone + oxytocin (n = 10). Differences are indicated as * p < 0.05; ** p < 0.01; ***p < 0.001.

To elucidate potential pro-arrhythmic effects of the individual compounds, short term variability of QTc - a marker for temporal heterogeneity of repolarization - was assessed [19]. Oxytocin alone, fluoxetine and risperidone as well as their combination with oxytocin significantly increased STV_QTc_ compared to baseline (Fig. 2A-B), indicating a pro-arrhythmic potential. While the combined treatment with antipsychotic/depressant drugs and oxytocin also induced an increased STV_QTc_ compared to baseline, no differences could be observed between fluoxetine and risperidone and their combinations with oxytocin (Fig. 2A-B).

### 3.2 Monophasic action potential recordings

Monophasic action potentials in isolated Langendorff-perfused rabbit hearts were used to determine *ex vivo* effects on cardiac repolarization in whole hearts.

#### 3.2.1 Monophasic action potential duration

**Fig. 3:**
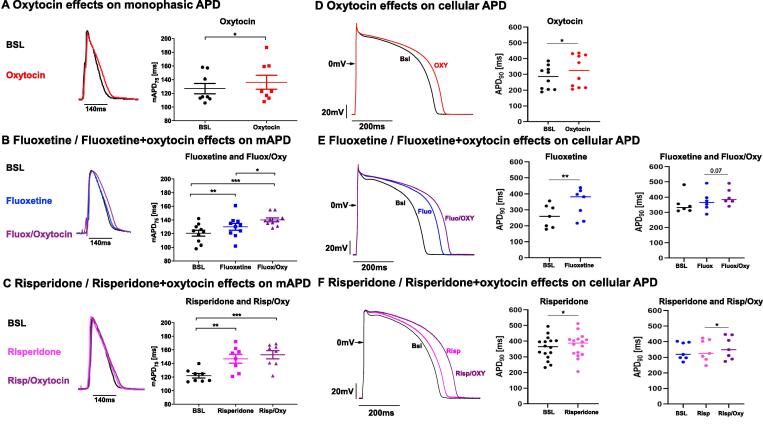
**Drug and hormone effects on APD in perfused hearts and isolated cardiomyocytes. A-C.** Effects on monophasic APD acquired in Langendorff-perfused hearts at 75% of repolarization (MAPD75) during perfusion with oxytocin (n = 8) (**A**), fluoxetine and fluoxetine + oxytocin (n = 10) (**B**), risperidone and risperidone + oxytocin (n = 8) (**C**) are shown. Exemplary monophasic action potential (AP) recordings are shown in left columns. Indicated are APs at baseline (black lines) and with hormones / drugs (colored lines). Middle column: Dots plots of individual APD_75_ at baseline and with hormone / drug perfusion. All monophasic APD were investigated at body temperature at a stimulation rate of 2 Hz. **D-F.** Drug and hormone effects on cellular action potentials recorded in current clamp. Representative examples of the prolonging effect of oxytocin (Oxy) (**D**), fluoxetine (Fluo) (**E**), risperidone (Risp) (**F**) and their combination with Oxy on action potentials recorded from rabbit cardiomyocytes at room temperature at a stimulation rate of 1 Hz. Control drug-free (Bsl) action potentials are indicated in black. Right columns show dots plots of individual APD_90_ at baseline and with hormone / drug perfusion. Differences are indicated as * p < 0.05; ** p < 0.01; ***p < 0.001.

Mean APD_75_ measured at 2 Hz stimulation frequency was significantly prolonged in oxytocin-perfused hearts compared to baseline (Fig. 3A).

Fluoxetine and fluoxetine + oxytocin also prolonged mean APD_75_ compared to baseline (Fig. 2B). Importantly, the combined treatment with oxytocin and fluoxetine significantly prolonged mean APD_75_ compared to fluoxetine alone (Fig. 3B) - but did not lead to a potentiation of the APD-prolongation.

Similarly, risperidone and risperidone + oxytocin increased mean APD_75_ compared to baseline (Fig. 3C). However only a trend towards a further prolongation could be observed by the combination of risperidone + oxytocin compared to risperidone alone.

### 3.3 Patch clamping

#### 3.3.1 Cellular action potential duration

Oxytocin (at 200 pg/ml) prolonged APD at 1 Hz pacing compared to baseline (Fig. 3D).

Fluoxetine (3 µM) also prolonged APD compared to baseline (Fig. 3E). In a subset of cells, in which a combination of fluoxetine + oxytocin was applied, fluoxetine + oxytocin slightly (but non-significantly) prolonged APD compared to baseline and the APD prolonging effect of combination of fluoxetine + oxytocin tended to be more pronounced than fluoxetine alone (*p = 0.07*, Fig. 3E).

Similarly, risperidone (1 µM) prolonged APD compared to baseline (Fig. 3F). In a subset of cells, in which a combination of risperidone + oxytocin was applied, risperidone + oxytocin also tended to prolong APD compared to baseline and, importantly, the risperidone + oxytocin combination significantly prolonged APD compared to risperidone alone (Fig. 3F).

None of the hormones/drugs affected resting membrane potential (RMP), action potential amplitude (APA) and the maximum velocity of depolarization (d*V*/d*t_max_*, V_max_)

#### 3.3.2 Underlying mechanisms - effects on repolarizing ion currents

**Fig. 4:**
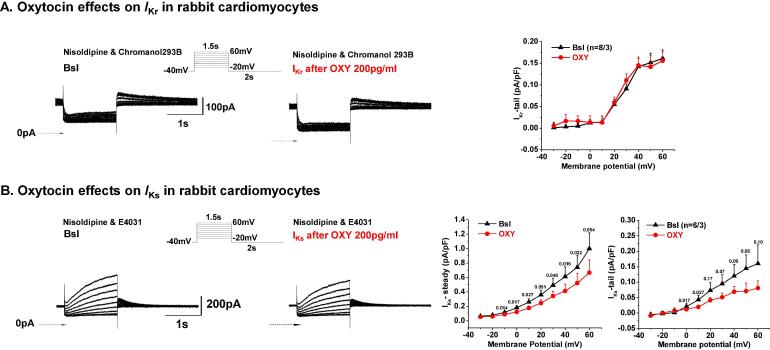
**Oxytocin effects on repolarizing I_Kr_ and I_Ks_ currents. A.** Effects of oxytocin (OXY) on I_Kr_ in rabbit cardiomyocytes. Left: Representative current traces of I_Kr_ currents before (left) and after 3-min application of 200 pg/mL OXY (right). Voltage (V_m_) protocol shown in inset. Horizontal arrows indicate zero current level. Right: IV-curve depicting mean peak I_Kr_-tail plotted as a function of the applied voltage. **B.** Effects of oxytocin (OXY) on I_Ks_ in rabbit cardiomyocytes. Left: Representative current traces of I_Ks_ measured before and after 200 pg/mL OXY perfusion. Voltage (V_m_) protocol shown in inset. Right: IV-curves depicting mean I_Ks_-end pulse and peak I_Ks_-tail plotted before (baseline) and after OXY as a function of the preceding test pulse potential.

To reveal the underlying ionic mechanisms of the prolongation in APD after risperidone, fluoxetine and their combination with oxytocin, we focused on studying the (total) delayed rectifier current (I_K_ - a composite of I_Ks_ and I_Kr_) since previous studies have indicated that risperidone and fluoxetine block I_Kr_ [5], [6], while we have demonstrated that oxytocin does not affect I_Kr_, but causes a significant decrease in I_Ks_ [15) (Fig. 4).

In Fig. 4, these effects of oxytocin are summarized: oxytocin did not alter I_Kr_-tail currents (Fig. 4B). In contrast, oxytocin significantly reduced I_Ks_-tail by approximately 47% at + 40 mV (Fig. 4A). The decrease in I_Ks_-steady (end-pulse) was less pronounced, approximately –33% at + 40 mV.

The electrophysiological effects of fluoxetine and risperidone in cardiomyocytes had been previously studied extensively - demonstrating direct I_Kr_-blocking properties in cardiomyocytes of various species and resulting APD- and QT-prolonging effects [20], [21], [22], [23], [24]. We therefore conducted measurements of fluoxetine and risperidone effects on I_Kr_ only in limited number of cells, in which we confirmed previous results regarding I_Kr_-blockade (Suppl. Fig. 2): Fluoxetine (3 µM) decreased I_Kr-_tail at + 40 mV by 47%. Risperidone (1 µM) decreased I_Kr-_tail at + 40 mV by 28%.

**Fig. 5:**
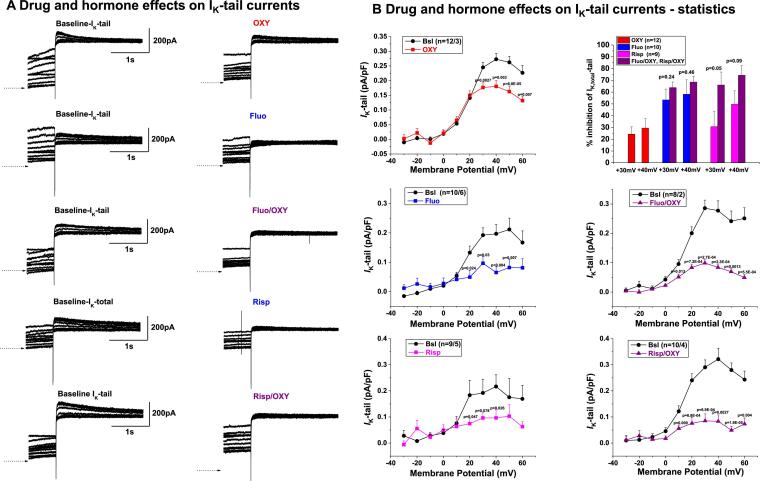
**Effect of hormones and drugs on the delayed rectifier potassium current (I_K_)** recorded in rabbit cardiomyocytes. **A.** Representative current recordings in control conditions (left) and in presence of OXY, Fluo + OXY and Risp + OXY (right). **B.** IV-curves depicting the current–voltage relationships for I_K_-tail currents under control conditions and in the presence of OXY and in the presence of the drugs with and without oxytocin. Right: Bar graphs indicating the % inhibition of I_K_-tail at + 30 mV and + 40 mV.

Oxytocin decreased I_Ktotal_-tail at + 40 mV by 34% (Fig. 5). Fluoxetine decreased I_Ktotal_-tail at + 40 mV by 67% (Fig. 5). When a combination of fluoxetine + oxytocin was applied, the inhibition was slightly bigger than fluoxetine alone (70% in I_K_-tail at + 40 mV Fig. 3). Risperidone alone blocked I_K_-tail at + 40 mV by 55.5% (Fig. 5). When a combination of risperidone + oxytocin was applied, the inhibition of I_K_-tail at + 40 mV was more pronounced than with risperidone alone (74%, Fig. 5), indicating the oxytocin-induced I_Ks_ block and the fluoxetine + risperidone induced I_Kr_ block are additive on repolarizing total I_K_.

The differential I_Ks_/I_Kr_-blocking effects of the compounds were also apparent when comparing their APD-prolonging effects at different frequencies. The I_Ks_ blocker oxytocin, exerted an APD-prolongation without frequency-dependence with similar APD prolongation at 0.5, 1, and 2 Hz (Suppl. Fig. 3A). Risperidone on the other hand behaved like a classical I_Kr_-blocker with reverse frequency-dependence [22] and caused a clear APD-prolongation at 1 Hz and no significant APD prolongation at 2 Hz (Suppl. Fig. 3B). Despite being also an I_Kr_-blocker (but also with other ion channel blocking properties) fluoxetine had no reverse frequency-dependence with similar APD-prolongation at 0.5 Hz and 1 Hz (Suppl. Fig. 3C) - as previously described [24], [25].

**Fig. 6:**
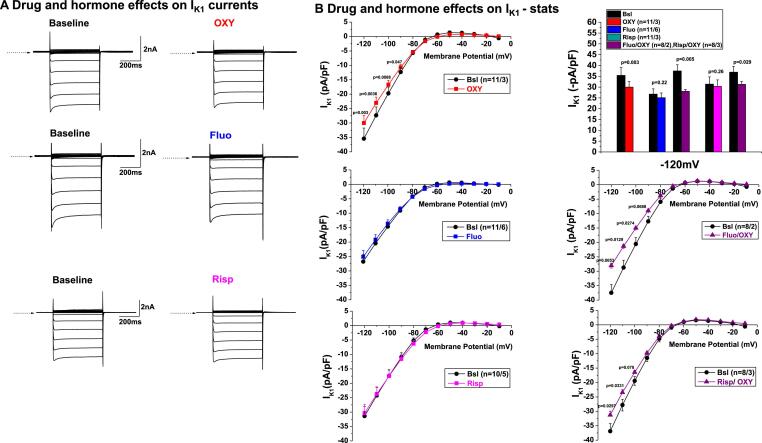
**Drug and hormone effects on I_K1_ in rabbit ventricular myocytes. A.** Representative examples of current recordings are shown in control conditions (left) and after application of hormone/drugs (right). Currents were elicited by test pulses between −120 mV and −10 mV from the holding potential of −20mV**B.** IV-curves depicting the current density–voltage relationships of peak components of *I_K_*_1_ shown in baseline conditions (black) and after exposure to the studied drugs (colored). Upper right panel: Bar graphs indicating the effects of OXY and Fluo, Risp and their combination with OXY on I_K1_ at −120 mV.

In addition, we investigated the potential electrophysiological effects on the inward rectifier potassium current (I_K1_) (Fig. 6). Oxytocin reduced I_K1_ at negative membrane potentials (from −120 to −90 mV, Fig. 6), thus further reducing the repolarization reserve in cardiomyocytes, while risperidone and fluoxetine alone had no effects on I_K1_ (Fig. 6). The combination of oxytocin with risperidone or with fluoxetine showed similar I_K1_ blocking effects as oxytocin alone (Fig. 6).

## 4 Discussion

Drug-induced LQTS remains an important and feared pharmacological side effect of a variety of drugs. This study demonstrated potentially harmful repolarization prolonging effects of the “binding-hormone” oxytocin, the anti-depressant fluoxetine, the anti-psychotic risperidone, and - particularly - their combination that is clinically used in various psychiatric disorders and elucidated the underlying mechanisms.

### 4.1 QT interval and other *in vivo* pro-arrhythmia markers

The evaluation of *in vivo* pro-arrhythmia markers such as QTc plays a major role in risk stratification for congenital and drug-induced LQTS [3], [26]. The combination of two or more QT-prolonging drugs is a known risk factor for drug-induced LQTS and resulting drug-induced pro-arrhythmia [27], [28]. However, this has mostly been demonstrated for the combination of several drugs blocking I_Kr_.

In this study, we demonstrated significant QTc prolonging effects of risperidone and fluoxetine as single drugs in wildtype rabbits, which is consistent with previous findings and case reports in human subjects [5], [23] - particularly following risperidone, while the QT-prolonging effects of fluoxetine seem to be more variable in individual patients [29], [30]. Likewise, oxytocin monotherapy significantly prolonged QTc in wildtype rabbits, similarly as in previous case reports and animal studies that have shown QTc-prolongation both in normal subjects and - more pronouncedly - in the setting of reduced repolarization reserve as in LQTS or in chronic-ischemia induced APD-prolongation [11], [31]. Along these lines, we recently demonstrated that oxytocin also prolongs QT and cellular APD in transgenic LQT2 rabbits and identified I_Ks_ blocking properties of oxytocin as the underlying electrophysiological mechanism [15].

We here demonstrated an additive effect of I_Ks_ blocking oxytocin and the anti-depressant fluoxetine or the anti-psychotic risperidone on QTc, causing more pronounced prolongation upon combined treatment compared to monotherapy. The term “repolarization reserve” indicates the ability of cardiomyocytes to maintain sufficient repolarization despite repolarization-prolonging effects by compounds due to a compensation via non-affected “reserve” K^+^ currents [32]. Risperidone and fluoxetine block I_Kr_ [5], [6]. The additional blockade of I_Ks_ through oxytocin thus leads to a further reduction of this repolarization reserve. This combination of a blockade of different ion currents can explain the enhanced prolongation of repolarization seen in this study.

While the question concerning an increased pro-arrhythmic effect of fluoxetine or risperidone monotherapy remains unanswered with contradictory evidence [3], [33] the combination with another QT-prolonging compound, such as oxytocin, could severely increase the risk for arrhythmia, especially by affecting different ion channels rather than the same. With the concept of the repolarization reserve in mind, a combination of risperidone or fluoxetine plus oxytocin reduces the repolarization reserve in such manner that arrhythmia development becomes more likely [27]. Important to note, however, only additive effects and no potentiation were seen during combined treatment, which may indicate a less pronounced pro-arrhythmic potential than in case of combined treatment with pure I_Kr_- and I_Ks_-blockers as in the classical experiments conducted by Biliczki et al. with dofetilide and chromanol [34].

In addition to QTc, we have investigated other markers of pro-arrhythmia: Beat-to-beat short-term variability of QTc (STVQT), which indicates temporal instability in cardiac repolarization and is a novel marker regarding risk stratification of cardiac arrhythmias. Increased STVQT is associated with higher risk for arrhythmia and could be used as a predictive marker [19]. In animal studies, alteration of STVQT was assessed both for I_Kr_ and I_Ks_ blockers, showing similar results in the extent of increase of STVQT [35], [36]. Especially the combination of both, I_Kr_ and I_Ks_ blockade, showed significantly increased and clinically relevant beat-to-beat variability [36]. Oxytocin, risperidone and fluoxetine significantly increased STV_QTc_, therefore underlining the pro-arrhythmic properties of these compounds and their combination.

Though we did not observe any spontaneous arrhythmias or early afterdepolarizations directly, we could previously demonstrate reduced repolarization reserve and increased propensity to pro-arrhythmic permanent depolarization, lack of 1:1 capture and early afterdepolarization formation in an *in silico* computational LQT2 model with lack of I_Kr_, in which we have incorporated the experimentally observed acute oxytocin effects [15]*.* Therefore, one may assume that in the context of acquired LQTS due to I_Kr_-blocking drugs (such as fluoxetine or risperidone) in combination with oxytocin, similar pro-arrhythmic events may occur.

### 4.2 Monophasic action potential duration in Langendorff-perfused hearts

Drug-induced prolongation of APD is considered a risk factor for the development of pro-arrhythmic early afterdepolarizations (EADs) and resulting arrhythmias [37]. On the whole heart level, risperidone / fluoxetine, and oxytocin prolonged monophasic APD - in line with the *in vivo* QT effects observed in our study. This observation is similar to previous studies [15], [33]. Moreover, we demonstrated that the combined treatment with oxytocin and fluoxetine significantly prolonged monophasic APD compared to fluoxetine alone and that for the combined treatment of risperidone and oxytocin a similar trend was observed, thus indicating additive effects of the combined treatment on cardiac repolarization - similarly as observed *in vivo*.

### 4.3 Underlying electrophysiological mechanisms

As demonstrated in heterologous HERG-expression systems and cardiomyocytes, risperidone is a potent blocker of I_Kr_ [5], [22], [23] and has a potent lengthening effect on cardiac repolarization (QT, APD) at clinically relevant drug concentrations [23]. Similarly, we confirmed I_Kr_-blocking effects of risperidone in rabbit cardiomyocytes and pronounced reduction of I_Kr_ and I_K_ total, which explain the observed QT- and APD-prolonging effects.

Similarly, fluoxetine - a multichannel blocker - also blocks I_Kr_ at clinically relevant concentrations [6] and thereby prolongs APD/QT. The repolarization prolonging effects are, however, not as pronounced as fluoxetine also inhibits the L-type calcium channel (I_Ca,L_) [24]. In our rabbit models, however, fluoxetine consistently prolonged QT and monophasic APD due to its effects on I_Kr_ and I_Ktotal_.

Oxytocin exerts its QT and APD prolonging effects via a different mechanism. As we have previously demonstrated, oxytocin does not affect I_Kr_ but reduces I_Ks_-steady and I_Ks_-tail currents [15]. Additionally, we could demonstrate here that oxytocin may also reduce I_K1_ at very negative membrane potentials (-90 to −120 mV), thereby further decreasing the repolarization reserve. The oxytocin-induced reduction of I_K1_ at very negative potentials will – theoretically – not directly prolong the QT/APD. The decrease of the inward component of I_K1_, however, may influence both active and passive membrane properties. Additionally, it could increase the resting membrane resistance together with membrane hypopolarization. Consequently, this could finally increase the likelihood of reaching threshold potentials, thus increasing the probability of AP discharge.

The additive effect of oxytocin and the anti-depressant fluoxetine or the anti-psychotic risperidone on cardiac repolarization (QT/APD) with more pronounced prolongation upon combined treatment compared to monotherapy can be explained by the different mechanisms via which the different drugs / hormones exert their effects on cardiac repolarization: risperidone and fluoxetine mainly block I_Kr_ [5], [6], and the additional blockade of I_Ks_ through oxytocin leads to a further reduction of the “repolarization reserve”.

Important to note, the effect of both risperidone and fluoxetine on cellular APD was less pronounced than the effects observed in the Langendorff-perfused hearts. This might be partially due to differences in the temperature at which cellular AP and MAP were acquired, as ion current characteristics are known to be affected by changes in temperature. Additionally, isolated cells are not coupled, while cardiomyocytes within the tissue are coupled to each other. The latter (more physiological situation) may change AP morphology due to the electrotonic sink, which again will indirectly change the relative contribution of individual ion currents [38] and thereby the effects of drugs on APD.

Indeed, similarly as in our cellular experiments, other studies observed very heterogenous effects on cellular APD ranging from clear APD prolongation to nearly no APD prolongation (or even sometimes APD-shortening by fluoxetine) in some preparations [5], [22], [24], [39], [40] – despite consistent I_Kr_-block - likely due to the additional I_Ca.L_ blocking effects of both drugs - which will lead to a shortening of APD and will counteract the block of risperidone and fluoxetine on I_Kr_, which otherwise would lead to a prolongation of APD in rabbit cardiomyocytes. Indeed, despite the pronounced inhibition of I_K_ by risperidone and fluoxetine that was observed in all rabbit cardiomyocytes investigated, in our experimental setting – similarly as in previous reports - in roughly 25% of isolated rabbit cardiomyocytes APD_50_ and APD_90_ was not prolonged after risperidone or fluoxetine treatment. This resulted in slight discrepancies between the extent of cardiac repolarization-prolonging effects between drug-induced QTc prolongation and cellular APD prolongation. For example, risperidone and risperidone + oxytocin prolonged QTc by 13% and 19%; while in patch clamp experiments, risperidone + oxytocin only prolonged cellular APD_90_ by 5%. Interestingly, in case of fluoxetine the QTc changes were comparable with APD_90_ data obtained in patch clamp experiments. Importantly, a similar %-prolongation of QTc (and APD) by risperidone and fluoxetine was also observed in other species such as cats and dogs [20], [22].

These data indicate that it is very important to also assess the effects of drugs or drug-combinations not only on cellular level, but also in the whole heart and the intact animal to fully investigate their potential pro-arrhythmic cardiac repolarization.

It is not yet known how oxytocin blocks KCNQ1/I_Ks_ on the molecular level. Theoretically, the effects could be exerted by direct blockade through an interaction with the channel protein or by an alteration of the KCNQ1/I_Ks_ expression via oxytocin-receptors. As the first I_Ks_ decreasing effects are seen within a very short time (∼2min) of oxytocin perfusion, a direct interaction seems to be plausible. Thus far, however, it is not known in which region of the channel protein this interaction may occur. In addition, we could previously demonstrate that changes in the expression of various channel occur in case of more chronic exposure - such as an increase in mRNA and protein levels of *CACNA1C/*Ca_v_1.2 and RyR2 - which may further aggravate acute effects on cardiac repolarization.

### 4.4 Clinical implications

Patients receiving more than one QT-prolonging drug bear an increased risk for cardiac arrhythmias. Administration of multidrug-treatment should be considered carefully especially in the context of drug-induced acquired LQTS. As this study suggests an additive effect on QT-prolongation when the “bonding-hormone” oxytocin - which has emerged as promising drug for a variety of psychiatric disorders - is combined with risperidone or fluoxetine, the indication for such a combined treatment should be considered carefully, and ECG / QT duration should be monitored regularly during treatment (particularly in situations in which additional pro-arrhythmic factors are present such as electrolyte imbalances). Important to note, however, only additive effects and no potentiation were seen during combined treatment, which may indicate a less pronounced pro-arrhythmic potential than in case of combined treatment with pure I_Kr_- and I_Ks_-blockers [34].

### 4.5 Limitations

Only adult female rabbits were used for the experiments, as both women and female rabbits are known to be more sensitive to I_Kr_-blocker induced drug-induced QT-prolongation than males [3]. Some studies indicate that QT-prolonging effects of oxytocin might be more pronounced in male rabbits [11]; hence the effect of oxytocin and combined treatment found in this study might differ between sexes.

Although rabbits show pronounced similarities with humans in cardiac electrophysiology [16] some species differences always exist, indicating that confirmatory studies in human subjects are warranted to evaluate the exact effect of combined treatment with oxytocin and psychiatric medication in human subjects. Additionally, the parameters investigated in this study are only indirect parameters for pro-arrhythmic properties of the drug combinations. Larger scale studies investigating the pro-arrhythmic effects of oxytocin, fluoxetine, risperidone and their combination with oxytocin directly need to be performed to further elucidate the clinical pro-arrhythmic potential of these drug combinations.

## 5 Conclusion

Treatment with multiple repolarization prolonging drugs is a main risk factor for patients to develop drug-induced acquired LQTS with potential malignant outcome. This study provides evidence that the hormone oxytocin prolongs QTc / APD and increases temporal heterogeneity of repolarization caused by I_Ks_ blockade. Combined treatment of the I_Ks_-blocking drug oxytocin with the antipsychotic I_Kr_-blocking drug risperidone or the antidepressant I_Kr_-blocking drug fluoxetine showed additional increase in QTc interval and APD, suggesting a potential harmful interaction in combined drug use.

## Funding Sources

German Research Foundation DFG OD 86/6-1 to K.E.O.

## Declaration of Competing Interest

The authors declare that they have no known competing financial interests or personal relationships that could have appeared to influence the work reported in this paper.
