# Supplementary material for: Oxytocin exerts harmful cardiac repolarization prolonging effects in drug-induced LQTS
Source: Int J Cardiol Heart Vasc. 2022 Apr 3;40:101001. doi: 10.1016/j.ijcha.2022.101001 (PMC8980310; doi:10.1016/j.ijcha.2022.101001)
Supplement: Supplementary data 1 [file mmc1.docx]

**Online supplement**

**Methods**

Animal studies

All animal experiments were performed in accordance with the German animal protection law (TierSchG) and the Directive 2010/63/EU of the European Parliament after approval by the local authorities (Regierungspraesidium Freiburg; protocol number G15/114).

The rabbit was chosen as model system due to pronounced species similarities in cardiac repolarizing ion currents between humans and rabbits (1) that facilitate future translation to human subjects. In this study, adult female wild type *New Zealand White* rabbits (average age 113 days for ECG and 125 days for MAP experiments) were used - as these are known to be more sensitive in respect to cardiac repolarization prolonging agents than male animals.

For sedation, a bolus of S-Ketamine (12.5 mg/kg, Pfizer, USA) and xylazine hydrochloride (3.75 mg/kg, Bayer, Germany) was administered i.m. for surface ECG and MAP experiments. Sedation was maintained by continuous i.v. infusion of ketamine/xylazine (3.0 ml/h) when performing surface ECG experiments, as this combination does not influence myocardial repolarization (2).

For monophasic action potential measurements beating hearts were excised after additional application of sodium heparin (1.000 IU, Braun, Germany) and sodium thiopental (40 mg/kg, Inresa, Germany).

Oxytocin, fluoxetin and risperidone were used at clinically relevant dosages.

Surface ECG

Surface ECG measurements were performed in sedated female WT rabbits at baseline and every two minutes for a period of twenty minutes after bolus injection of oxytocin (1.5 IU i.v., Sigma-Aldrich, USA), followed by continued oxytocin infusion for the duration of the recording 1 IU at a rate of 6 ml/h). Similarly, recordings with fluoxetine were performed in sedated female WT rabbits at baseline and every two minutes for a period of twenty minutes after bolus injection of fluoxetine (1 mg/kg i.v., Sigma-Aldrich USA), followed by fluoxetine infusion for the duration of the recording (infusion with 2 mg/ml at a rate of 4.5 ml/h). Twenty minutes after the fluoxetine bolus, a bolus of oxytocin (1.5 IU i.v) was applied and infusion with oxytocin for the following twenty minutes was started (infusion with 1 IU/ml at a rate of 6 ml/h). Measurements occurred again every two minutes for a period of twenty minutes. The same protocol was used for risperidone (0.3 mg/kg i.v. bolus, infusion with 0.3 mg/ml at a rate of 10 ml/h, Sigma-Aldrich, USA). I.v.-injection was used to ensure adequate blood concentration of the different compounds and to avoid uncertainties in serum concentrations following oral administration that may stem from species differences in pharmacokinetics. All medications used in these experiments relate to clinical used dosages in patients (3–5).

To ensure no residual effects of the applied drugs for those rabbits that were used for multiple surface ECG experiments, time intervals between the recordings were at least four half-lives.

RR-interval, QT duration, heart-rate corrected QT-index, QTi = QT/ (86 + 0.22*RR) (6), heart-rate corrected QTc (using Fridericia formula) (7) and short-term variability of the QT (STV_QT_) (8), as a marker for temporal heterogeneity of repolarization, were assessed. The Fridericia-correction formula was used for heart-rate correction as this adequately corrects the QT at the heart rates observed in these experiments, e.g., 120-140/min (9). For STV_QT_, 31 consecutive QT were measured and STV_QT_ was calculated using the following equation: STV_QT_ = ∑|D_n+1_−D_n_| (30×√2) ^−1^, where D is the duration of the QT intervals.

Monophasic action potential measurements (MAP)

To assess action potential duration, monophasic action potential measurements (MAP) were performed. First, rabbits were anesthetized using S-ketamine and xylazine as described above. After injection of heparine (1.000 IU i.v., Braun, Germany) the rabbits were euthanized with i.v. injection of thiopental-sodium (40 mg/kg i.v., Inresa, Germany). Thoracotomy was performed, the beating hearts were excised and immediately mounted at a Langendorff perfusion setup (Model IH5, Hugo Sachs Electronic, Harvard Apparatus GmbH, Hugstetten, Germany). Modified Krebs-Henseleit-buffer, heated (37 °C) and oxygenated with carbogen, was used as a blood substitute. For initial stimulation an external pacing electrode at the base of the heart was used with a basic cycle length of 500 ms (equal to a heart frequency of 120 beats per minute).

Atria were mechanically removed, and the AV node was ablated. Constant heart frequency of 120 bpm in ECG recording through external stimulation was considered as a successful ablation as the intrinsic AV node frequency exceeds the external pacing frequency of 120 bpm. To imitate a physiological filling of the ventricle, a latex balloon was inserted into the left ventricle.

Monophasic action potentials were assessed by using five epicardial contact MAP electrodes at five different cardiac locations. MAP1-4 was positioned from the apex to the base of the left ventricle, whereas MAP 5 represented the base of the right ventricle. The exact positions were MAP1: apico-anterior, MAP2: mid-anterolateral, MAP3: base-inferolateral, MAP4: base-inferior of the left ventricle and MAP5: base-inferior of the right ventricle.

After an equilibration time of about 20-30 min baseline measurements were performed at 2 Hz (cycle length of 500 ms), 3 Hz (CL 333 ms) and 4 Hz (CL 250 ms) stimulation frequency. Oxytocin (200ng/l), fluoxetine (3 µM) or risperidone (1 µM) was then administered in Krebs-Henseleit-buffer. Following perfusion with drug eluted Krebs-Henseleit-buffer for eight minutes to ensure building up a medication level, measurements at 2 Hz, 3 Hz and 4 Hz were performed the same way as described above. Finally, oxytocin was added to fluoxetine or risperidone respectively, and the same protocol as described was applied. For analyzing and recording of the action potential duration Isoheart® Software Version 1.1.1.128 was used (Hugo Sachs Electronic, Harvard Apparatus GmbH, Hugstetten, Germany).

Patch Clamping

***Isolation of rabbit ventricular cardiomyocytes***

Ventricular myocytes from LV wall were obtained from the hearts of WT rabbits (2-5kg, 4-7 and 13-15 months, male and female) by standard collagenase digestion. Rabbits were anesthetized with a combination of ketamine S (12.5mg/kg) and xylazine (3.75mg/kg) IM. After administration of heparin (1000UI/kg) and thiopental-sodium (~40mg/kg) IV, the hearts were rapidly excised and placed in ice cold Tyrode’s isolation solution (composed of in mM: 135 NaCl, 20 taurine, 4.4 NaHCO3, 4.7 KCl, 1.2 MgSO4, 10 HEPES, 10 glucose, 5 Na-Pyruvate, 1.2 KH_2_PO_4_, pH adjusted to 7.2-7.3 with NaOH) (10) mounted on a Langendorff apparatus and Tyrode solution supplemented with 1 mM Ca^2+^ was perfused through the aorta - (3-4 min at 25-30mL/min). Perfusion was then switched to nominally Ca^2+^-free isolation solution containing 96 µM EGTA (5-10 min; 15mL/min), and then digestion was performed with Ca^2+^-free solution supplemented with 0.8-1 mg/mL collagenase (Worthington type 2, 305 U/mg; Worthington Biochemical Corp., Freehold, NJ) and 33 µM Ca^2+^ for 25-40 min. All perfusates were gassed with 100% O_2_ and maintained at 37°C. At the end of the digestion, the heart was then removed from the cannula, the atria and right ventricle were cut off and discarded. The left ventricle was gently teased apart in Krafte-Brühe solution (KB solution) containing (in mM): KOH 89, KCl 30, HEPES 10, EGTA 0.5, Glucose 11, Taurine 15, L-Glutamic acid 70, MgCl_2_ 0.5, KH_2_PO_4_ 10 pH adjusted to 7.3 with KOH) to disperse single cells (11). Subsequently, the dissociated cells were filtered and washed twice with KB solution by centrifuging at a speed of 500rpm for 3 min. The supernatant was discarded and the cell pellet was resuspended in KB solution and was stored at ~4-8°C. The experiments were performed within 6-8hrs of isolation. Only quiescent, rod-shaped myocytes with clear cross striations and no evidence of membrane blebbing were selected for patch-clamp studies.

***Electrophysiological recording in rabbit cardiomyocytes***

To assess the effects of oxytocin and its combination with the psychopharmaceutic drugs on AP and ion currents patch clamp experiments were performed.

Whole cell currents and action potentials (AP) were recorded using Axopatch 200B patch clamp amplifier (Molecular Devices, Sunnyvale, California), digitized at a sampling frequency of 10kHz with Digidata 1440A interface and acquired with pCLAMP software (version 10.2, Axon Instruments). Action potentials (AP) were recorded under current clamp, holding at 0 pA. APs were elicited by square-wave stimulus pulses of 5 ms in duration at 50% above the threshold intensity (2-4 nA) and a cycle length of 1s (1Hz), and in some cases a cycle length of 0.5s (2Hz) and 2s (0.5Hz). AP was measured at steady state, defined as last of the train of 15 cardiac cycles at the same stimulation rate. Patch electrodes were pulled from BRIS Micro hematocrit tubes (Iso 12772, Vitrex Medical A/S, Denmark) using a gravity puller (Model PC-10, Narishige Corp.). Pipette resistance ranged from 1.5-2.6mΩ when filled with the internal pipette solution containing in (mM): 90 K-aspartate, 30 KCl, 5.5 glucose, 1.0 MgCl_2_, 5 EGTA, 5 Mg-ATP, 5 HEPES, 10 NaCl, pH adjusted to 7.2 with KOH. All experiments were performed at 20-23°C. Ventricular cells were placed in a transparent perfusion chamber (Warner Instruments) mounted on the stage of an inverted microscope (Leica) and allowed to settle for ~5min.

Membrane currents were recorded using the whole cell configuration of the patch clamp technique. For studies of I_K_ (I_Kr_, I_Ks_), and I_K1_ cardiomyocytes were superfused continuously at 1-2mL/min with normal Tyrode solution containing (in mM): 126 NaCl, 5.4 KCl, 1.0 MgCl_2_, 2.0 CaCl_2_, 10 HEPES, and 11 Glucose, pH adjusted to 7.4 with NaOH (12). To measure I_Kr_, I_Ca,L_, and I_Ks_ were inhibited by 1µM nisoldipine (N0165-Sigma), and 30µM chromanol 293B (C2615-Sigma), respectively. *I-V* relationship for I_K_ and I_Kr_ tail currents were determined by applying 1.5s depolarizing voltage pulses from holding potential of -40mV to test potentials ranging from -30 to + 60mV at an interpulse interval of 5s (0.2Hz). Tail current was measured following different test potentials upon repolarization to -40mV (2.0s).

I_K1_ was elicited from a holding potential of -20mV by voltage steps of 500ms from -120mV to +50mV in 10mV increments every 5s. This protocol was repeated in presence of 2mM BaCl_2_. I_K1_ is given as the Ba^2+^-sensitive current (13). Steady-state I_K1_ amplitudes were estimated at the end of the 500 ms pulse.

Cell membrane capacitance was calculated for each cell by integrating the area under the uncompensated capacity transient elicited by a 25 mV hyperpolarizing test pulse (25ms) from a holding potential of 0mV. Individual currents were normalized to the membrane capacitance to control for differences in cell size and expressed as current density (pA/pF). Experiments were discarded when the series resistance was high (>9 MΩ) or increasing during the experiments. All experiments were carried out at room temperature. Due to the number of different drugs used and hence the time needed to perform the experiments the authors chose to carry out all experiments at room temperature. This ensured more stable data acquisition compared to performing the experiments at physiological room temperature.

In the present study, three conditions were used to minimize I_Ks_ run-down as described in the literature (14,15): small tip electrodes (3-5 mOhm) with short shanks, a negative pressure was maintained inside the electrode, and the bath temperature was RT (22-23C). This way, cell dialysis was minimized and changes in osmolarity due to possible K^+^ leakage from the pipette into the cell can be negligible. However, even under these conditions, I_Ks_ run-down cannot be completely prevented. To ensure that the results obtained are accurate, cells with > 40% run-down prior to the drug application were excluded. Important to note, the results of I_Ks_ block in cardiomyocytes is very much in line with previous recording in heterologous expression systems (KCNQ1-KCNE1 stably expressed in CHO cells), in which the well-established (Amphotericin B) perforated configuration to prevent run-down of I_Ks_ was used (16), indicating that with our approach, we were able to reduce run-down quite well. In cardiomyocytes, especially for action potential recordings (using current clamp), the perforated patch clamp technique is often used in the literature to minimize the wash-out of cytosolic components. However, this protocol has not yet been established in rabbit cardiomyocytes in our lab.

Data were analyzed using Clampfit, version 10.6 and Origin 8.1 software (Microcal Software, Northampton, CT, USA) was used for graphical data analysis. To analyze the action potentials, the Matlab program, Edition 2017a was used.

Oxytocin (oxy) stock solution was dissolved in distilled water (stock solutions: 1mg/mL) and stored at -20°C. At the day of each experiment stock solutions were diluted to required concentrations (200pg/mL) in recording solution. Risperidone was diluted from 10mM stock solution (dissolved in DMSO). Fluoxetine was also diluted from 10 mM stock (dissolved in H_2_O).

Statistics

Data are expressed as mean ± standard error (SEM). Statistical analyses were performed by [Prism](https://scicrunch.org/resources/Any/search?q=SCR_005375%26l=SCR_005375) 8.0 ([Graphpad](https://scicrunch.org/resources/Any/search?q=SCR_000306%26l=SCR_000306), San Diego, USA) Microsoft Excel 2016 (Microsoft corporation, Redmond, Washington, USA), and Prism StatMate. Graphs were created by Prism 8.0. Comparisons between groups were performed using unpaired t-tests and one-way ANOVA. Comparisons between values recorded before and after hormone / drug administration within the same groups were carried out using 2-tailed paired Student’s t tests. The acceptable maximal α error was set at 5%. All data shown normal distribution, therefore, no non-parametric test had to be done.

**References**

1. Nerbonne JM. Molecular basis of functional voltage-gated K+ channel diversity in the mammalian myocardium. J Physiol. 2000 Jun 1;525(Pt 2):285–98.

2. Odening KE, Hyder O, Chaves L, Schofield L, Brunner M, Kirk M, et al. Pharmacogenomics of anesthetic drugs in transgenic LQT1 and LQT2 rabbits reveal genotype-specific differential effects on cardiac repolarization. Am J Physiol Heart Circ Physiol. 2008;295(6):H2264–72.

3. Cochran D, Fallon D, Hill M, Frazier JA. The role of oxytocin in psychiatric disorders: A review of biological and therapeutic research findings. Harv Rev Psychiatry. 2013;21(5):219–47.

4. Jannuzzi G, Gatti G, Magni P, Spina E, Pacifici R, Zuccaro P, et al. Plasma Concentrations of the Enantiomers of Fluoxetine and Norfluoxetine: Sources of Variability and Preliminary Observations on Relations With Clinical Response. Therapeutic drug monitoring. 2002 Nov 1;24:616–27.

5. Kent JM, Kushner S, Ning X, Karcher K, Ness S, Aman M, et al. Risperidone Dosing in Children and Adolescents with Autistic Disorder: A Double-Blind, Placebo-Controlled Study. J Autism Dev Disord. 2013 Aug 1;43(8):1773–83.

6. Brunner M, Peng X, Liu GX, Ren X-Q, Ziv O, Choi B-R, et al. Mechanisms of cardiac arrhythmias and sudden death in transgenic rabbits with long QT syndrome. J Clin Invest. 2008;118(6):2246–59.

7. Fridericia LS. The duration of systole in an electrocardiogram in normal humans and in patients with heart disease. Acta Med Scand. 1920;8(4):343–51.

8. Hinterseer M, Thomsen MB, Beckmann B-M, Pfeufer A, Schimpf R, Wichmann H-E, et al. Beat-to-beat variability of QT intervals is increased in patients with drug-induced long-QT syndrome: a case control pilot study. Eur Heart J. 2008;29(2):185–90.

9. Vandenberk B, Vandael E, Robyns T, Vandenberghe J, Garweg C, Foulon V, et al. Which QT Correction Formulae to Use for QT Monitoring? J Am Heart Assoc. 2016 Jun 17;5(6).

10. Major P, Baczkó I, Hiripi L, Odening KE, Juhász V, Kohajda Z, et al. A novel transgenic rabbit model with reduced repolarization reserve: long QT syndrome caused by a dominant‐negative mutation of the KCNE1 gene. Br J Pharmacol. 2016 Jun;173(12):2046–61.

11. Farkas A, Acsai K, Nagy N, Tóth A, Fulop F, Seprényi G, et al. Na + /Ca 2+ exchanger inhibition exerts a positive inotropic effect in the rat heart, but fails to influence the contractility of the rabbit heart. British journal of pharmacology. 2008 Jun 1;154:93–104.

12. Dumaine R, Cordeiro JM. Comparison of K+ currents in cardiac Purkinje cells isolated from rabbit and dog. J Mol Cell Cardiol. 2007 Feb;42(2):378–89.

13. Rose J, Armoundas AA, Tian Y, DiSilvestre D, Burysek M, Halperin V, et al. Molecular correlates of altered expression of potassium currents in failing rabbit myocardium. Am J Physiol Heart Circ Physiol. 2005 May;288(5):H2077-2087.

14. Sanguinetti MC, Jurkiewicz NK. Two components of cardiac delayed rectifier K+ current. Differential sensitivity to block by class III antiarrhythmic agents. J Gen Physiol. 1990;96(1):195–215.

15. Balser JR, Bennett PB, Hondeghem LM, Roden DM. Suppression of time-dependent outward current in guinea pig ventricular myocytes. Actions of quinidine and amiodarone. Circ Res. 1991 Aug;69(2):519–29.

16. Bodi I, Sorge J, Castiglione A, Glatz SM, Wuelfers EM, Franke G, et al. Postpartum hormones oxytocin and prolactin cause pro-arrhythmic prolongation of cardiac repolarization in long QT syndrome type 2. Europace. 2019 Jul 1;21(7):1126–38.

**Supplemental Figures**


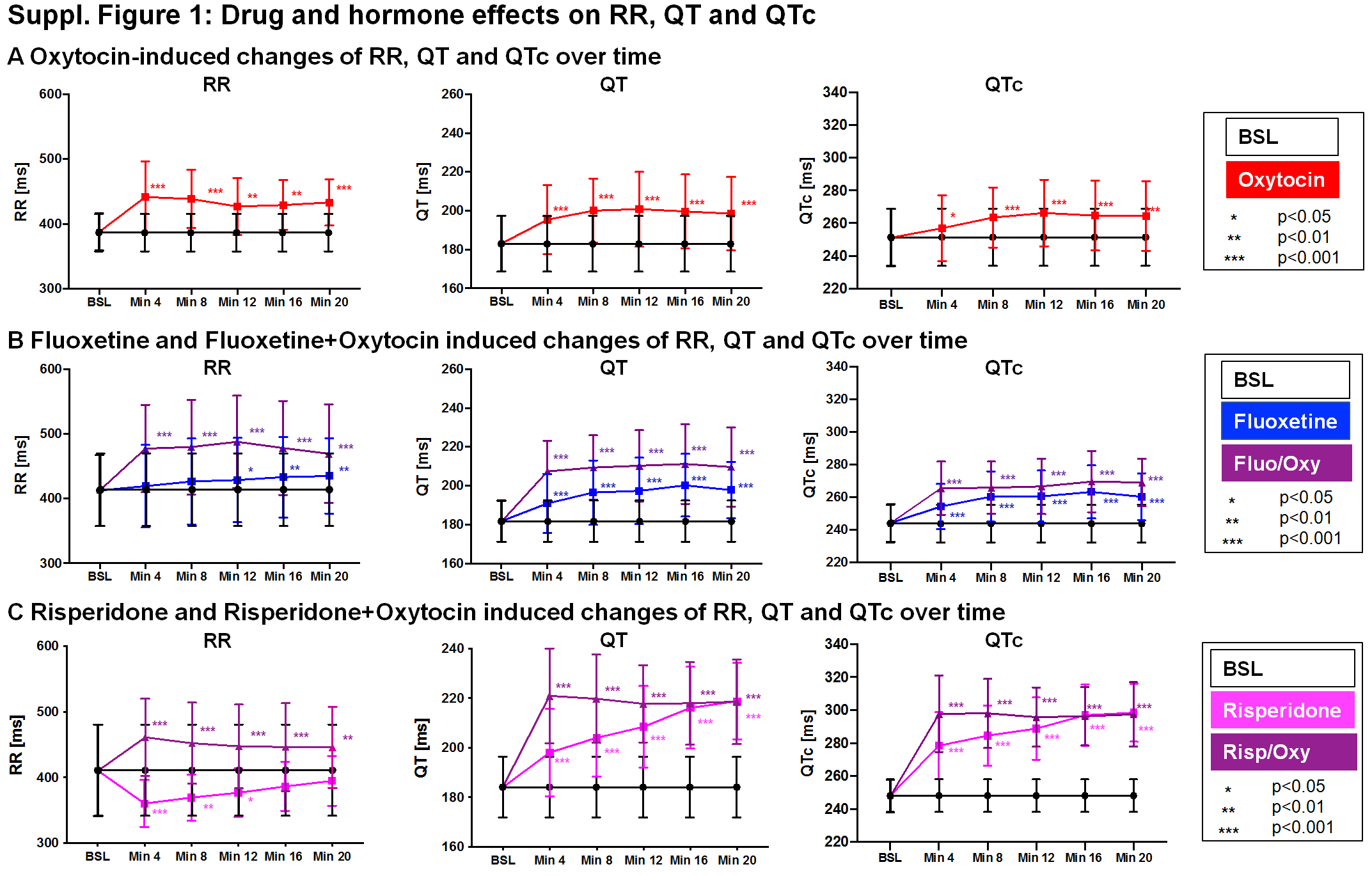


**Suppl. Figure 1: Drug and hormone effects on QT, RR and QT-index in surface ECG.** Indicated are changes over time in QT, RR, and QTc during perfusion with oxytocin (n=13) (**A**), fluoxetine and fluoxetine+oxytocin (n=23) (**B**), risperidone and risperidone+oxytocin (n=21) (**C**).

**
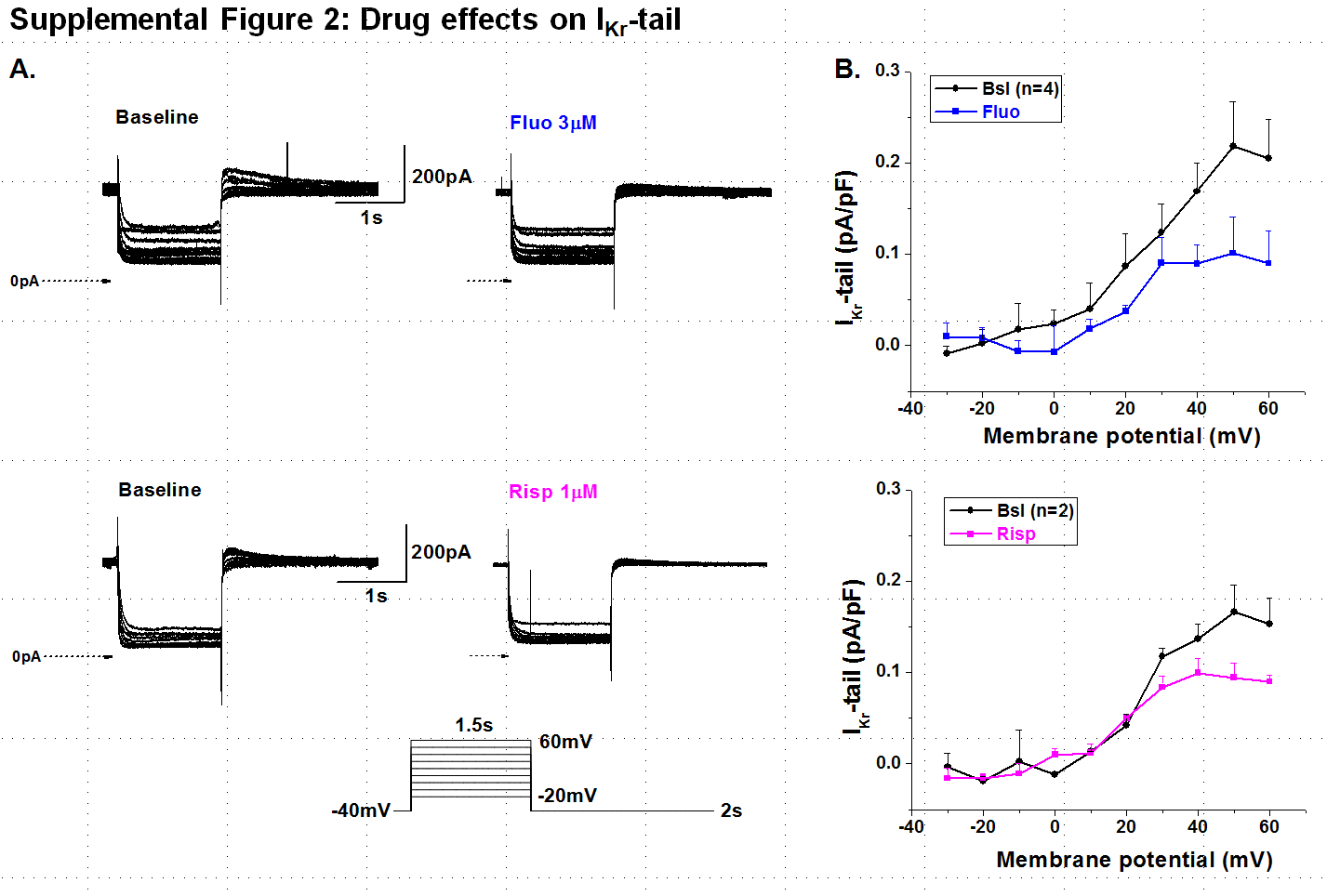
**

**Supplemental Figure 2: Drug effects on I_Kr_-tail. A.** Representative I_Kr_ current tracings at baseline and with fluoxetine (Fluo, 3µM) or risperidone perfusion (Risp, 1µM). **B.** IV-curves of I_Kr_-tail at baseline and with fluoxetine (Fluo, 3µM) or risperidone perfusion (Risp, 1µM) indicating a significant reduction of I_Kr_ by both drugs.


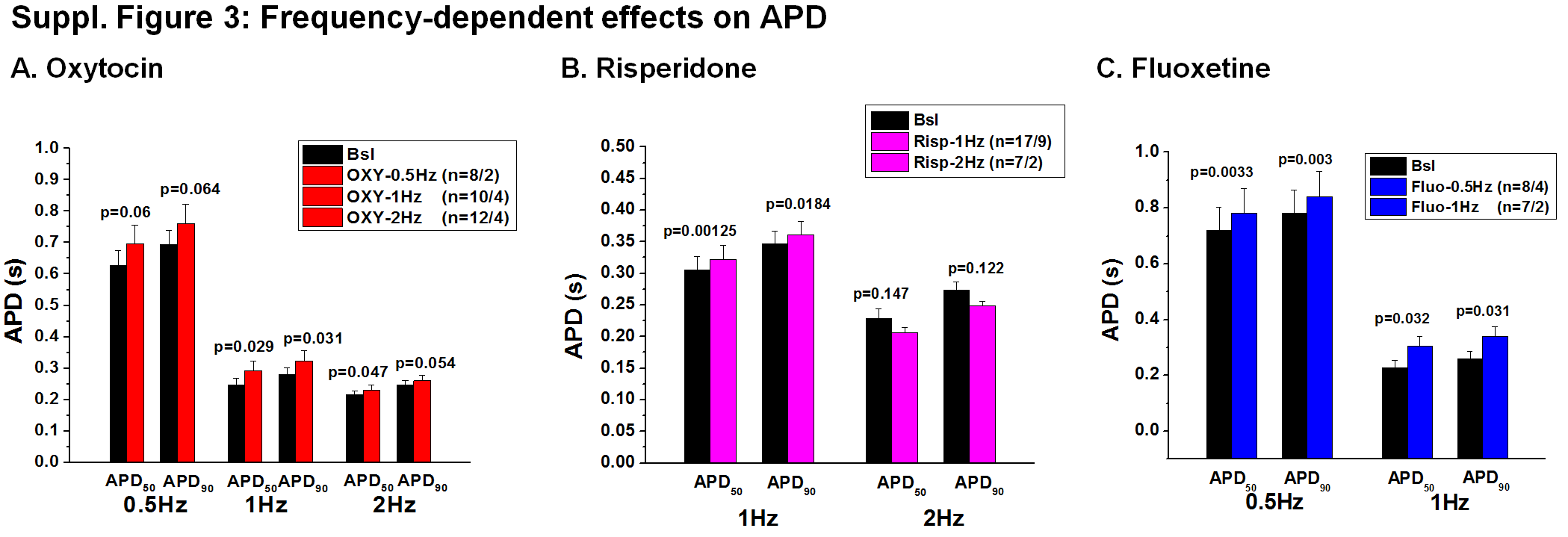


Supplemental Figure 3: Frequency-dependent effects on APD. **A.** Oxytocin effects on APD_90_ and APD_50_ at 0.5 Hz, 1 Hz, and 2 Hz stimulation. **B.** Risperidone effects on APD_90_ and APD_50_ at 1 Hz and 2 Hz stimulation. **C.** Fluoxetine effects on APD_90_ and APD_50_ at 0.5 Hz and 1 Hz stimulation.
